# Varicella-zoster virus susceptibility and primary healthcare consultations in Norway

**DOI:** 10.1186/s12879-016-1581-4

**Published:** 2016-06-07

**Authors:** Grazina Rimseliene, Kirsti Vainio, Moustafa Gibory, Beatriz Valcarcel Salamanca, Elmira Flem

**Affiliations:** Norwegian Institute of Public Health, Infection Control and Environmental Health, PO Box 4404, NO-0403, Oslo, Norway

## Abstract

**Background:**

Currently Norway does not recommend universal varicella vaccination for healthy children. This study assessed susceptibility to varicella-zoster virus (VZV) in the Norwegian population for the first time.

**Methods:**

A national convenience sample of residual sera was tested for anti-VZV IgG by ELISA. We estimated age-specific seropositivity to VZV, controlling for sex and geographical distribution. We assessed differences between the proportions using the chi-square test and multivariable logistic regression. Seroprevalence data were compared to the varicella and herpes zoster-associated consultation rates in patients attending primary healthcare.

**Results:**

Although 73.2 % (*n* = 1,540) of all samples were positive for VZV, only 11.2 % of samples collected from 1-year-olds were seropositive. There was a sharp increase in the proportion of seropositive in 3- and 5-year-olds (40.2 % and 65.4 %, respectively). By the school entry age of 6 years, 69.8 % of children were seropositive. The age-specific annual consultation rate for varicella in primary healthcare peaked in 1-year-olds, with 2,627 cases per 100,000 population. The profile of varicella-related consultations in primary healthcare mirrored the VZV seropositivity profile. The herpes zoster-related consultations in primary healthcare peaked in people over 70 years of age (702 cases per 100,000 population).

**Conclusions:**

VZV seroprevalence in Norway was somewhat lower than in some other European countries. The age-specific varicella–related consultation rates in primary healthcare mirrored the age profile of VZV seroprevalence.

**Electronic supplementary material:**

The online version of this article (doi:10.1186/s12879-016-1581-4) contains supplementary material, which is available to authorized users.

## Background

Varicella-zoster virus (VZV) is a ubiquitous DNA virus that belongs to the Herpesviridae family. The virus spreads via airborne droplets and direct contact [1] and causes varicella (chickenpox) and herpes zoster (shingles; HZ) [1]. Varicella is a contagious childhood disease that is usually benign [1]. However, an estimated 2–6 % of varicella cases that seek care from a clinician develop complications such as bacterial superinfections or neurologic or pulmonary disorders [1, 2]. Although such complications can occur in previously healthy children, the risk is higher for adults [1]. The virus also establishes latency in the neurons of sensory ganglia [3] and later, in association with diminished VZV-specific cell-mediated immunity, may reactivate causing HZ [4]. The lifetime risk for HZ from natural infection is estimated to be 25 %, with most cases occurring in people over 50 years of age and in immunocompromised individuals [5].

Safe and effective varicella vaccines have been available since the 1970s [6], and vaccine against HZ is available since 2006 [7]. Recently, a new candidate vaccine against HZ has been developed as well [8]. However, despite recommendations from the World Health Organization (WHO) [9, 10] and the European Working Group on Varicella [11], only some European countries have integrated the varicella vaccine into national immunization programs [12, 13]. There is a concern that universal varicella vaccination may result in an increased incidence of HZ due to the possible decline of exogenous boosting following a reduced circulation of the wild type virus [14]. In addition, high vaccination coverage is needed to avoid shifting varicella morbidity to older age groups [10]. In Norway, varicella vaccine is not currently offered through the national immunization program, but it is recommended for non-immune individuals [15] and is fully reimbursed for those who are at risk of complications, such as people with immunodeficiencies and stem cell transplantation patients [16]. Otherwise, the vaccine is available at a cost per dose of 465 Norwegian kroner (NOK) [17], which is roughly equivalent to $57 USD. Based on the National Immunization Registry SYSVAK, approximately 550 doses of varicella vaccines are given to 450 individuals annually in Norway, with a birth cohort of 60,000 children per year. There is currently no national recommendation regarding the use of the HZ vaccine [18]. This vaccine has been licensed in Norway since 2006 and is available at a cost of 1,748 NOK or roughly $220 USD per dose; approximately 100 doses have been sold since its licensure.

The availability of the varicella and HZ vaccines highlights the urgent need to assess the public health burden of these diseases in Norway in order to inform national vaccine policy decisions. Such an evaluation should be supported by the assessment of VZV seroprevalence in the population to understand the age-dependent dynamics of the infection and to identify susceptible groups. In Norway, few data about VZV seroepidemiology are available. All earlier studies focused on subsets of the population, such as patients with multiple sclerosis, infectious encephalitis, or pregnant women of foreign descent [19–21]. Therefore, we examined the anti-VZV antibody levels in different age groups in a sample of the Norwegian population and identified population groups with the lowest immunity against VZV. We also compared seropositivity proportions with the age-specific consultations rates for varicella and HZ in patients attending primary healthcare.

## Methods

### Study design and data sources

This was a cross-sectional seroprevalence study conducted using anonymized residual sera collected from patients of all ages who sought either primary or hospital care in Norway. Because all samples were anonymized, reasons for healthcare visits and associated sample collection are unknown. Laboratories however exclude samples from known HIV and hepatitis cases. Sera specimens are collected from all 19 counties throughout Norway during a 5-week period each year, usually in July–August. This study used residual sera obtained in 2006, 2007, 2008, 2011, and 2014 and excluded samples collected during the influenza pandemic of 2009–2010. The following information was available for each sample: patient birth year, sex, county of residence, sample collection date, and laboratory name.

The sample size in the study for each age group was calculated using a 95 % confidence interval (95 % CI) with a 10 % margin of error. As a result, roughly 100 samples were selected for each of the following age groups: 1-year bands between 0 and 9 years; 5-year bands between 10 and 49 years; 10-year bands between 50 and 69 years; and 100 samples from those 70 years old and older. These age groups were chosen to allow comparisons with data from other European countries.

The sera were stored at −20 °C at the Norwegian Institute of Public Health where the testing was performed. IgG antibody levels were measured using a commercial indirect enzyme-linked immunosorbent assay (ELISA); Enzygnost anti-VZV-IgG Virus/IgG, Siemens Healthcare Diagnostics AS, Erlangen, Germany) with the automated EVOLIS™ System from Bio-Rad and the DS2 Processing System from DYNEX. According to the manufacturer, the sensitivity of this method is 99.3 % and the specificity is 100 %. The assay was run in accordance with the manufacturer’s instructions. The positive and negative controls from the kit were used to validate the assay and results. We had no kit independent controls available. The cut-off for qualitative evaluation of positivity was a corrected optical density (OD) >0.2 at 450 nm. Samples with ODs <0.1 were counted as negative, and samples with ODs between 0.1 and 0.2 were considered equivocal. Equivocal samples were not re-tested.

The rates of primary healthcare consultations associated with varicella and HZ were measured using health reimbursement data from 2008–2012 extracted from the Norwegian Health Economics Administration database. The database includes individual reimbursement claims from all primary care providers in Norway. The extracted data included all consultations that had varicella or HZ at any diagnostic position, coded as A72 and S70, respectively, according to the International Classification of Primary Care, Second Edition (ICPC-2). The age- and sex-specific rates per 100,000 population were calculated using the number of primary care patients registered with varicella and herpes zoster diagnoses for the first time during 2008–2012 as the nominator and population data for the same time period as the denominator [22].

### Data analysis

VZV seropositivity was estimated as a proportion with the corresponding 95 % CI. We used the chi-square test to examine differences in seropositivity by age, sex and geographical regions. We also performed multivariable logistic regression analysis to assess the association between VZV seroprevalence, which was classified as positive or negative, and a set of explanatory variables (sex, age, geographic region). We assessed the fit of the different models using likelihood ratio tests. Statistical significance was set at a P-value <0.05. All analyses were performed using the statistical software STATA, version SE13 (StataCorp LP, College Station, TX, USA).

## Results

**Fig. 1:**
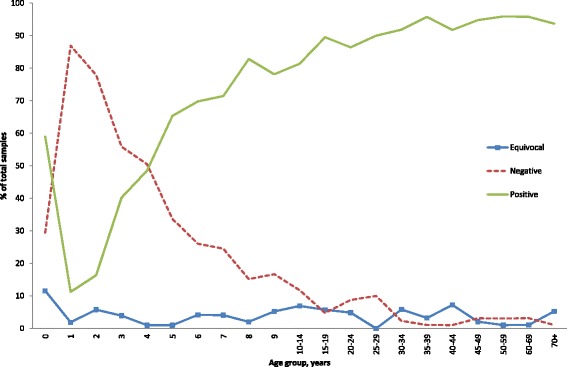
Age-specific varicella-zoster virus seroprevalence as measured by serum IgG antibodies among a subset of Norwegian population (*n* = 2,103)

A total of 2,103 samples from patients aged 0 to 92 years were included in the study, 51.9 % (*n* = 1,093) of which were from males. Overall, 73.2 % (*n* = 1,540) of the samples were seropositive for VZV (Table 1). The proportions of seropositive males and females were similar, 50.6 % and 49.4 %, respectively. The seropositivity proportion in children under 1 year of age was 58.9 %. This decreased to 11.2 % at the age of 1 year, likely reflecting a short-lived immunity conferred by maternal antibodies [23, 24]. The proportion of seropositive individuals increased to 40.2 % and 65.4 % at 3 and 5 years of age, respectively. By school entry age, which is 6 or 7 years old, 69.8 % and 71.4 % of children, respectively, were immune to varicella. The proportion of immune children increased further to 81.4 % by age 10–14 years (Fig. 1). By age 20 years, 86.4 % of the Norwegian population had acquired natural varicella immunity, and by age 35–39 years, 95.7 % of subjects had detectable anti-VZV antibodies.

**Table 1:** Age-specific varicella-zoster virus seroprevalence (%, 95 % CI) among a subset of Norwegian population (*n* = 2,103)

Age group	Positive		Negative		Equivocal	
	% (No of samples)	95 % CI	% (No of samples)	95 % CI	% (No of samples)	95 % CI
0 y	58.9 (56)	48.8–68.4	29.5 (28)	21.1–39.4	11.6 (11)	6.5–19.8
1 y	11.2 (12)	6.5–18.8	86.9 (93)	79.1–92.1	1.9 (2)	0.5–7.2
2 y	16.3 (17)	10.4–24.8	77.9 (81)	68.9–84.9	5.8 (6)	2.6–12.3
3 y	40.2 (41)	31.1–50.0	55.9 (57)	46.1–65.2	3.9 (4)	1.5–10.0
4 y	48.5 (49)	38.9–58.2	50.5 (51)	40.8–60.2	1.0 (1)	0.1–6.8
5 y	65.3 (66)	55.5–74.0	33.7 (34)	25.1–43.5	1.0 (1)	0.1–6.8
6 y	69.8 (67)	59.8–78.2	26.0 (25)	18.2–35.8	4.2 (4)	1.6–10.6
7 y	71.4 (70)	61.7–79.5	24.5 (24)	17.0–34.0	4.1 (4)	1.5–10.4
8 y	82.8 (82)	74.0–89.1	15.2 (15)	9.3–23.7	2.0 (2)	0.5–7.8
9 y	78.1 (75)	68.7–85.3	16.7 (16)	10.4–25.5	5.2 (5)	2.2–12.0
10–14 y	81.4 (118)	74.2–86.9	11.7 (17)	7.4–18.1	6.9 (10)	3.7–12.4
15–19 y	89.5 (94)	82.0–94.1	4.8 (5)	2.0–11.0	5.7 (6)	2.6–12.2
20–24 y	86.4 (89)	78.3–91.8	8.7 (9)	4.6–16.0	4.9 (5)	2.0–11.2
25–29 y	90.0 (81)	81.8–94.7	10.0 (9)	5.3–18.2	0 (0)	-
30–34 y	91.9 (79)	83.8–96.1	2.3 (2)	0.6–8.9	5.8 (5)	2.4–13.3
35–39 y	95.7 (90)	89.1–98.4	1.1 (1)	0.1–7.2	3.2 (3)	1.0–9.5
40–44 y	91.8 (89)	84.3–95.8	1.0 (1)	0.1–7.0	7.2 (7)	3.5–14.4
45–49 y	94.8 (91)	88.0–97.8	3.1 (3)	1.0–9.3	2.1 (2)	0.5–8.0
50–59 y	95.9 (94)	89.6–98.5	3.1 (3)	1.0–9.1	1.0 (1)	0.1–7.0
60–69 y	95.8 (91)	89.3–98.4	3.2 (3)	1.0–9.4	1.1 (1)	0.1–7.2
70+ y	93.7 (89)	86.6–97.1	1.1 (1)	0.1–7.2	5.3 (5)	2.2–12.1
Total	73.2 (1540)	71.3–75.1	22.7 (478)	21.0–24.6	4.0 (85)	3.3–5.0

Females of childbearing age, defined as those aged 15 to 49 years old [25], accounted for 34 % of all samples collected from women (343/1,010) and for 16.3 % of all tested samples. Of these samples, the overall seropositivity proportion was 88.6 %. The average proportion of seronegative females in this age group was 5.3 %. The proportion of non-immune women was highest, 13 %, in young adulthood (20–24 years); this proportion declined in the older age groups.

We also assessed the VZV seroprevalence in seven geographic regions defined by the population density [22]. The seropositivity proportions ranged from 59 % in sparsely populated central Norway to 86 % in densely populated Southeast Norway. However, multivariable analysis indicated that age group was the only explanatory variable that was significantly associated with VZV seropositivity (Additional files 1 and 2).

**Fig. 2:**
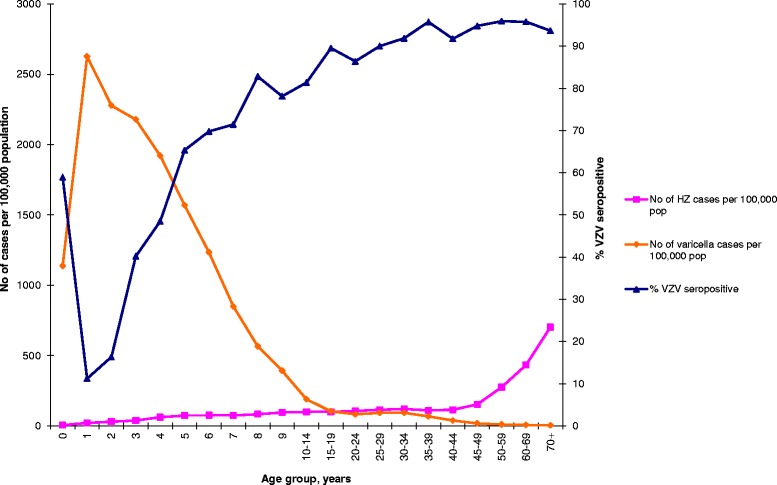
Age-specific varicella-zoster virus seropositivity (serum IgG antibodies) and age-specific consultation rate (cases per 100,000 population) of varicella and herpes zoster as first encounter with primary healthcare in Norway 2008–2012. The blue line represents percent of VZV seropositive individuals as measured by serum IgG antibodies in a subset of Norwegian population (*n* = 2013). Orange line shows the number of varicella cases per 100,000 population measured as first encounter with primary healthcare in Norway, 2008–2012 (*n* = 56,126). The pink line represents number of herpes zoster cases per 100,000 population, measured as first encounter with primary healthcare in Norway, 2008–2012 (KUHR)

From 2008–2012, there were a total of 73,065 varicella-related primary healthcare consultations by 56,134 persons in Norway, corresponding to an average annual consultation rate of 231 cases per 100,000 population. The highest consultation rate, 2,627 cases per 100,000 population, was observed in children aged 1 year; the lowest consultation rate was found in patients ≥70 years old. The varicella consultation rate in primary healthcare mirrored the VZV seroprevalence profile (Fig. 2), with children under 10 years old accounting for 79.3 % of all varicella cases. The majority of varicella patients (80 %) had only one encounter in a primary healthcare setting, mostly with GP (75 %). In 2008–2012, there were 124,139 HZ consultations by 54,094 persons in Norway, translating to an average annual rate of 223 cases per 100,000 population. The highest HZ consultation rate was observed in patients ≥70 years old (702 cases per 100,000 population), and the lowest rate was found in children in their first year of life (5.6 cases per 100,000 population). Most HZ patients had one or two encounters (76 %) with primary healthcare, and the majority (80 %) were GP consultations.

## Discussion

This is the first study to describe the age-specific seroprevalence of anti-VZV antibodies in different age groups in a Norwegian population. Because the varicella vaccine is currently used infrequently in Norway, we documented the pre-vaccine seroepidemiology of VZV and the use of primary healthcare associated with varicella and HZ. Overall, 73.2 % of the Norwegian population has natural immunity against varicella, with the highest seropositivity, 95.7 %, found in adults 35–39 years of age, suggesting almost universal transmission of VZV infection. Varicella-related consultation rates in primary healthcare mirrored VZV seroprevalence, with a peak in children aged 1 year. This pattern suggests a possible correlation between these different measures of varicella occurrence. For HZ, the opposite pattern was observed, prompting further investigation of the factors that influence the occurrence of this disease in Norway.

Similar to other European countries, varicella immunity in Norway is acquired gradually, starting in early childhood and showing a sharp increase around age 3–5 years. By this age, 90 % of Norwegian children have entered organized childcare [26], thereby increasing their opportunities for varicella exposure. By the school entry age of 6 years, 7 of 10 children are already immune, and an additional 10 % acquire natural immunity by age 10 years. The latter is somewhat lower than findings in other Nordic countries. For comparison, 91 % of children are reported to be seropositive by age 10 years in Finland [27], and in Sweden, 98 % of 9–12-year-olds are immune to varicella [28]. This is higher than the 78 % found in the same age group in our study, and there is no clear explanation for the difference. The 12 % of susceptible individuals aged 10–14 years in Norway is higher than the 8.3 % found in the same age group in Spain [27] or in Poland, where 82 % are seropositive by the age of 10 years [29]. We also found somewhat higher proportions of susceptibility in young children and adolescents compared to other countries, e.g. England and Wales, Belgium, Israel, Ireland, Netherlands, Slovakia [27], and Poland [29]. In Europe, VZV seroprevalence differs by country. Although, a standardized VZV seroprevalence study in 11 European countries demonstrated that over 90 % of children are VZV seropositive by age 15 years in most of the countries [27], for 5-year-old children, the lowest proportions of seropositive individuals were found in Italy (38 %) [27] and Poland (48 %) [29], and the highest proportion was found in the Netherlands (95–97 %) [30].

In our study, the proportion of susceptible adults aged 20–29 years was 9.5 %, whereas in most other European countries, this proportion is less than 5 %, except in Italy (11.5 %), the UK (7.1 %), Spain (6.9 %), and Ireland (6.2 %) [27]. Among females of childbearing age (15–49 years) in Norway, the proportion of non-immune subjects was 5.3 %. Nardone et al. reported such proportion to be less than 5 % in most European countries, except for Ireland (5.4 %) and Italy (12.5 %) [27]. The results of Nardone et al. are not directly comparable to our findings due to their use of a slightly different age group (15–39 years). It is difficult to compare our results with countries that were not included in the study by Nardone et al. due to methodological differences and variations in the age groups.

The level of IgG antibodies in a single sample may vary in different assays. A high percentage of equivocal samples detected in young adults in our study may therefore partly be due to the assay chosen for the study, for which international standards were not used.

Differences in VZV seropositivity levels in different European countries can be explained in part by varying population densities and social mixing patterns in the countries and perhaps by climate differences. However, it is surprising that VZV seropositivity in the children and adults in our study was somewhat lower than in reports from other Nordic countries with comparable populations and climates. It is possible that our results were somewhat affected by the convenience sampling used in the study. Such sampling is subject to selection bias because residual samples are collected from people seeking medical help, limiting the generalizability of the results. Despite these limitations, this method is often preferred in seroepidemiological studies over more generalizable population-based probability sampling. Convenience sampling is less costly and time-consuming, and the samples are easier to obtain [31]. Moreover, the VZV seroprevalence as estimated by convenience sampling is shown to be similar to the results of studies that use population-based cluster sampling [32]. To increase study validity, we collected samples from all geographic regions in the country and selected sera only from large microbiological laboratories that test patients who receive both primary and hospital healthcare. All residents in Norway have universal access to healthcare, so it is possible that our data included individuals who visited a healthcare provider for prophylactic purposes.

We found high seropositivity in infants (58.9 %), but this dropped sharply to 11.2 % by age 1 year, which may be explained by waning maternal antibodies [23, 24, 33]. In children aged one year, the proportion of seropositive subjects in our study was similar to the proportions in Finland, Italy, and Spain. However, in the majority of other European countries, the proportions are higher, varying between 20 % and 40 % [27]. Nevertheless, the actual age at sample collection in our study was not available; thus, the year of birth was subtracted from the year of sample collection, which could have affected the results for children under one year of age. Given that samples are collected in July–August of each year, the age group that was under one year of age in our sample was composed of children aged 0 to 8 months, whereas the one-year age group included children aged 7 to 20 months. This age distribution could result in overestimation of seropositivity in those under one year of age and underestimation of seropositivity in those older than one year. With increasing age, this difference would not have such a dramatic effect on seropositive proportions. However, this could be only verified if the actual age was reported rather than just the year of birth.

Our sample size was estimated to allow a detailed assessment of seroprevalence in children because we expected high seropositivity in adults. Although the total number of samples in our study (*n* = 2,103) was similar to the number of samples used in other European studies, we had fewer samples per age group in adults compared to, for example, the study by Nardone et al. (100 vs. 200 samples per 5-year age band) [27, 34]. Since we used anonymized sera, we cannot determine the number of samples that were collected from people who originate from tropical and subtropical countries, where lower varicella immunity in adolescents and young adults is established [35]. Since the 1990s, the estimated proportion of people of foreign descent in Norway has increased to 13 %, of which about one-third (26 %) originate from Asia [22]. A similar pattern is reported in Sweden [36] but not in Finland (3 %) [37], suggesting that the higher seroprevalence among Swedish children compared to our findings could be due to the timing of sample collection. Samples in Sweden as well as in Finland were collected in 1997–1998 when the proportion of immigrants was considerably lower than in 2006 and later when there were higher proportions of people of foreign descent in Norway. Therefore, the probability of samples being collected from people originating from settings that have shown differences in their varicella epidemiology may be higher in Norway than in other Nordic countries. In the Netherlands, being a foreign national was associated with lower VZV seropositivity in children under 6 years old [30], and more seronegative adults were found to originate from tropical and subtropical countries [35, 38]. Since ethnicity data were not available for this study, this hypothesis requires further research.

The differences between geographical regions found in our study should be interpreted with caution. Sampling bias may minimize the study’s power to find differences since the study was designed primarily to measure VSV seroprevalence in different age groups on the national level, not on the regional level. We found that 5.3 % of women of childbearing age were susceptible to VZV. This proportion was even higher in those aged 20–24 (13 %) and 25–29 years old (11 %). This is a potential concern because women in these age groups give birth to 45 % of the infants born annually in Norway. VZV infection during pregnancy can lead to serious complications, such as maternal pneumonia and congenital varicella syndrome [1]. However, these findings should also be interpreted with caution because they, too, could be affected by sampling bias. Since women born outside of Norway may lack immunity to varicella, more evidence is needed to define the groups for pregnancy screening in order to reduce the risk of potential VZV complications.

Overall, the primary healthcare consultation rate of both varicella and HZ was lower in Norway than in other European countries [34]. However, a direct comparison with other studies is very difficult due to differences in methodology as well as varying VZV epidemiology from country to country. We found a similar varicella consultation rate in Canada in a study by Brisson et al. [39] that use a similar data source (the physician billing claims); however, that study used data from 1979–1997. The consultation rate in our study was calculated using information on reimbursement claims from primary care providers. It is thus unlikely that the consultation rate was greatly underestimated because healthcare is generally easily accessible to all Norwegian residents. However, it may be somewhat underestimated, as patients with mild symptoms, and for example additional family members with infection may not seek medical help. In addition, the disease could be misclassified due to atypical presentation. We observed a peak in the consultation rate of varicella-related primary care consultations around 1 year of age. This is the age at which children are likely to be susceptible due to loss of protection conferred by maternal antibodies, and this is supported by our seroprevalence curve. However, it is difficult to determine whether this increase represents a true increase in the varicella incidence in the general population or whether this peak reflects an increase in more severe cases that have complications requiring medical help. It is also possible that the increase is affected by healthcare-seeking behavior since parents may be more likely to seek medical help when a young child contracts varicella.

A high proportion of susceptibles in certain age groups detected in our study underlines a need to revise Norwegian varicella vaccine recommendations such as expanding current recommendations to include adolescents aged 10–15 years without a positive history of varicella. There is also a need to consider varicella screening in pregnancy to identify non-immune women to be targeted for vaccination after giving birth. Such recommendations may potentially reduce the proportion of susceptible individuals at older ages and reduce the risk of complications.

## Conclusions

The VZV seroprevalence in Norway was somewhat lower than in some other European countries. The age-specific varicella-related consultation rates in primary healthcare mirrored the age profile of VZV seroprevalence. These data lay the ground for further research to quantify the disease burden of varicella and HZ and predict the impact of potential vaccination programs through mathematical modeling.

## Abbreviations

ELISA, enzyme-linked immunosorbent assay; HZ, Herpes zoster; IgG, immunoglobulin G; VZV, varicella-zoster virus.

## Additional files

Additional file 1: Univariate and multivariable analysis of varicella-zoster virus seropositivity by sex (reference: female), age group and region (reference: Oslo and Akershus) in Norway (odds ratios (OR), 95 % confidence intervals (95 % CI), and p-value). (XLSX 12 kb) Additional file 2: Model fitting information (Likelihood ratios and 95 % confidence intervals (CI)). (S: Sex; R: region; A: age groups. Df: the number of parameters that differ between the two nested models; AIC: Akaike information criterion). (XLSX 10 kb)
